# Supplementary figures and images for: Ataxia telangiectasia and Rad3-related inhibition by AZD6738 enhances gemcitabine-induced cytotoxic effects in bladder cancer cells
Source: PLoS One. 2022 Apr 12;17(4):e0266476. doi: 10.1371/journal.pone.0266476 (PMC9004738; doi:10.1371/journal.pone.0266476)

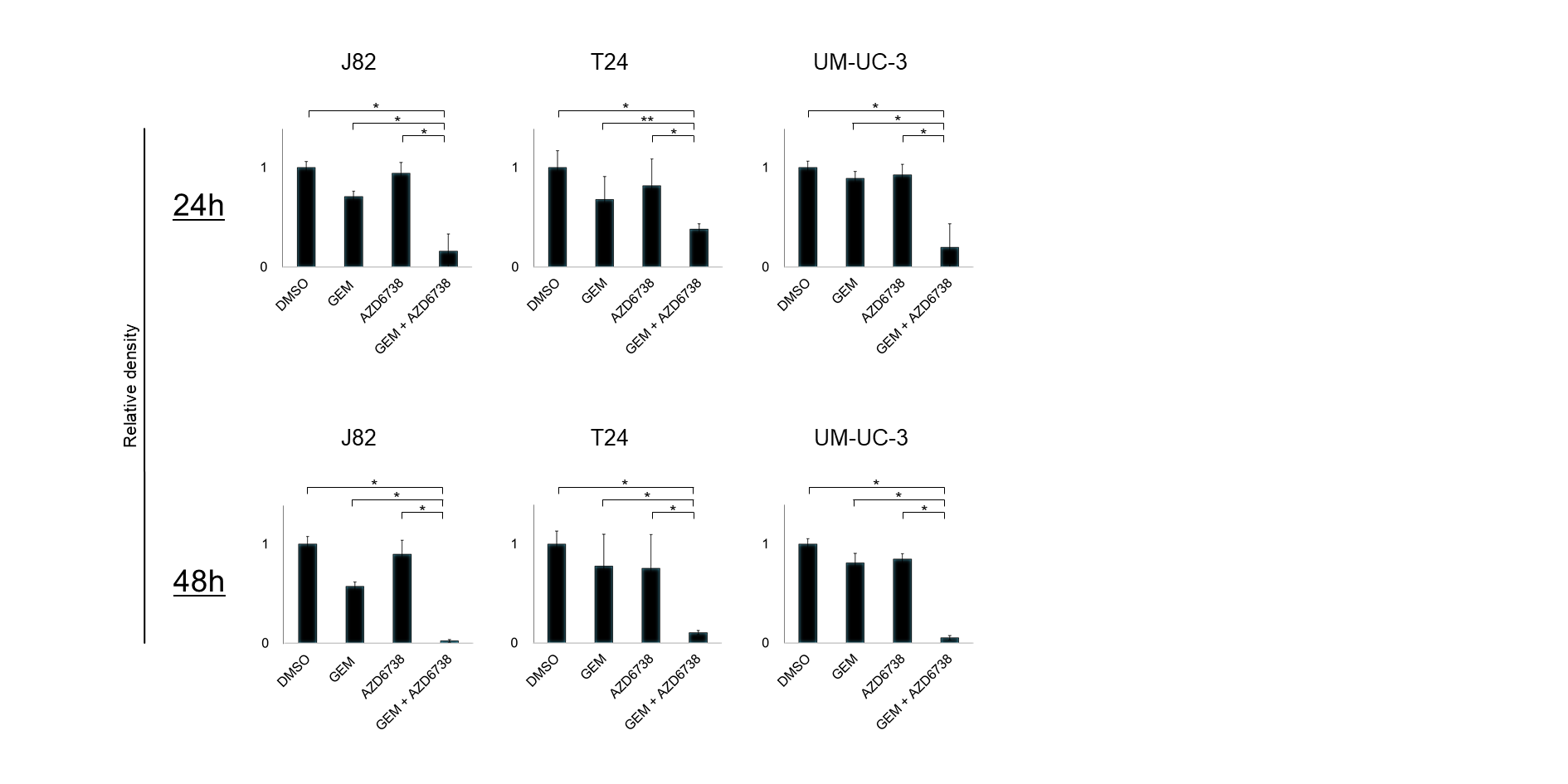

Supplement: S1 Fig — DMSO was used as negative control. Bar graphs show the relative density of the cells at each treatment. *p < 0.05, **p > 0.05. (TIF) [file pone.0266476.s001.tif]

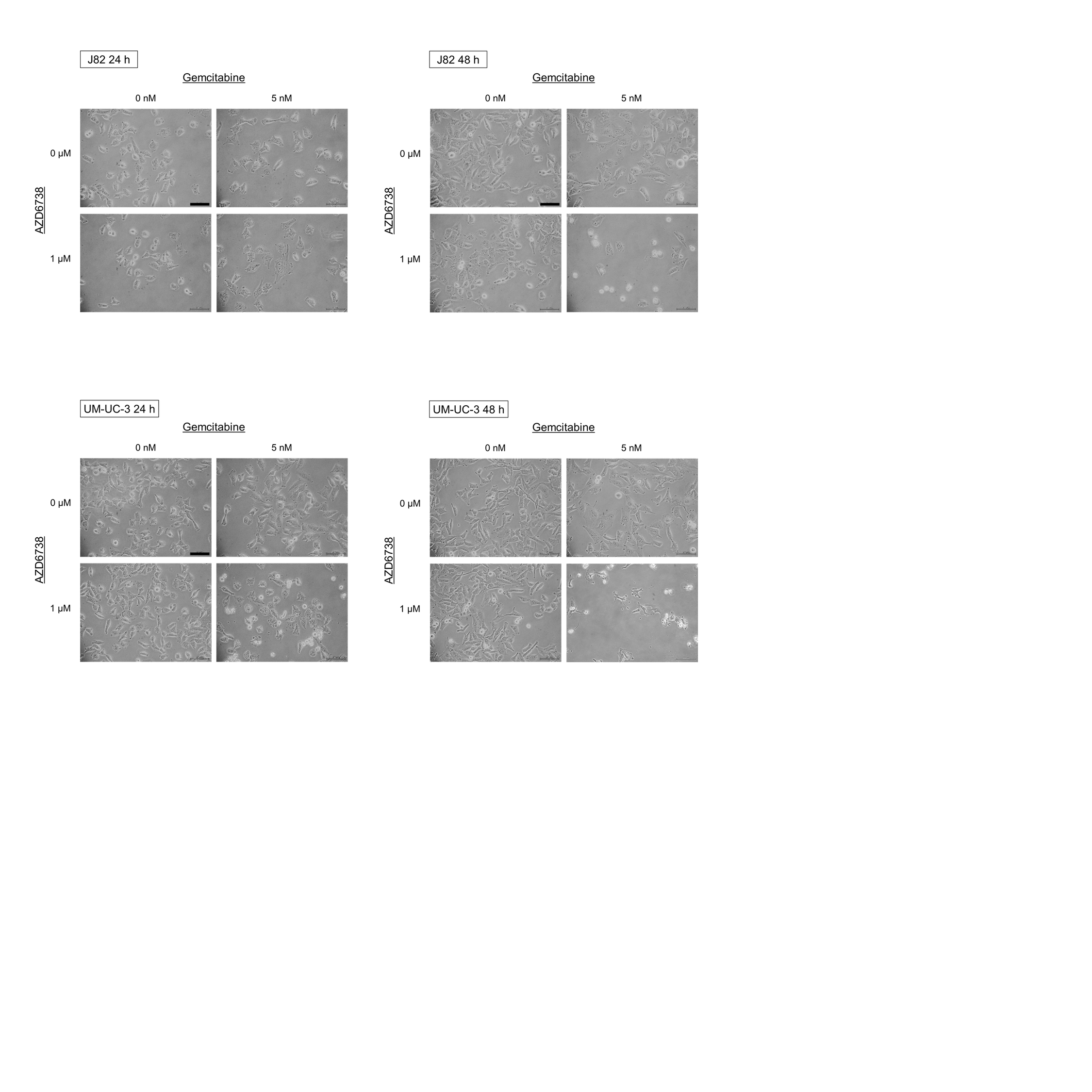

Supplement: S2 Fig — Scale bar: 100 μm. (TIF) [file pone.0266476.s002.tif]

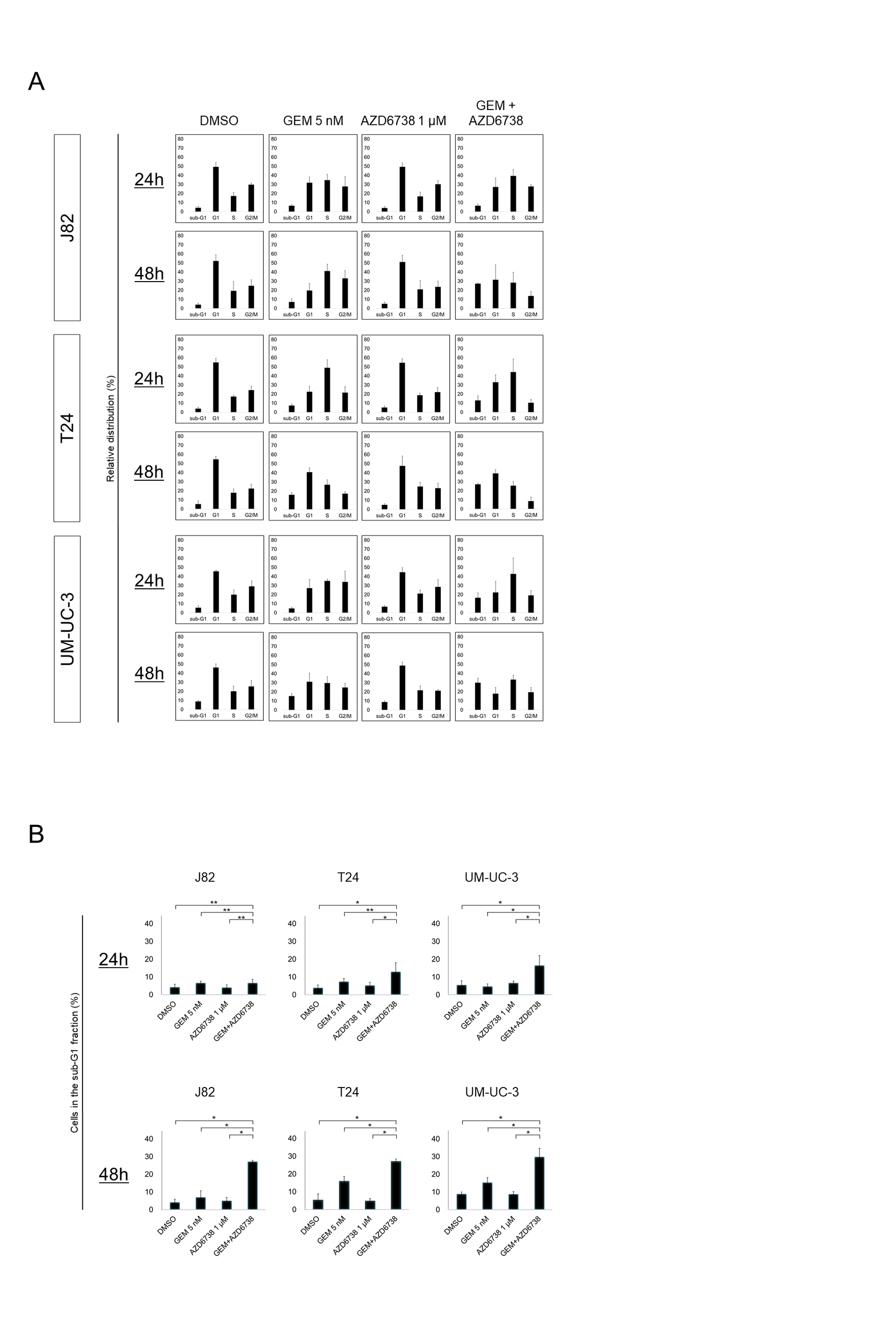

Supplement: S3 Fig — DMSO was used as negative control. (A) Bar graphs show the relative distribution of the cells at each phase of the cell cycle. (B) Bar graphs show the percentages of the cells in the sub-G1 fraction. *p < 0.05, **p > 0.05. (TIF) [file pone.0266476.s003.tif]

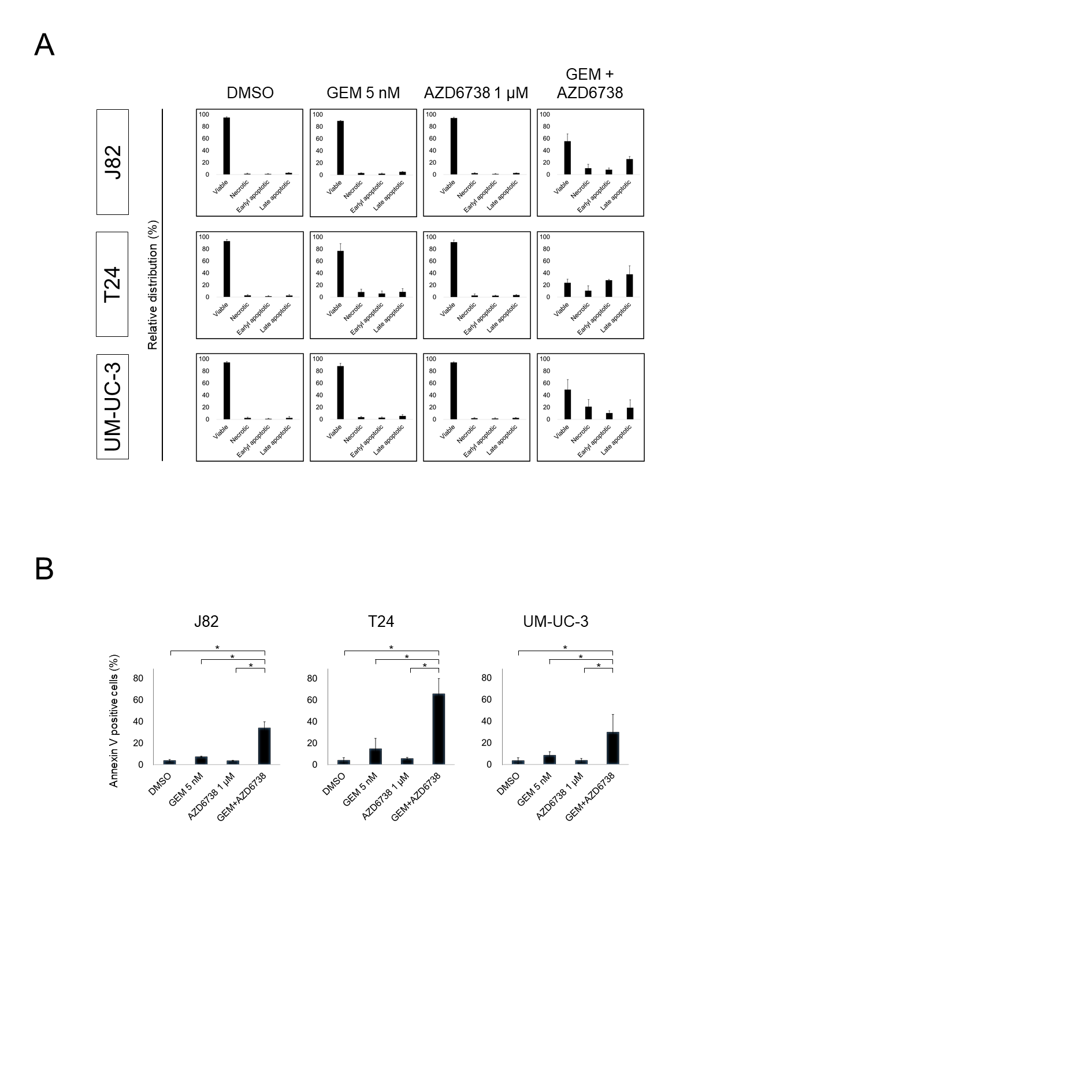

Supplement: S4 Fig — (A) The results are expressed as a percentage of early apoptotic cells, late apoptotic cells and necrotic cells. Bar graphs show the relative distribution of the cells at each quadrant. (B) Bar graphs show the percentages of apoptotic cells. *p < 0.05. (TIF) [file pone.0266476.s004.tif]

Figure 2B

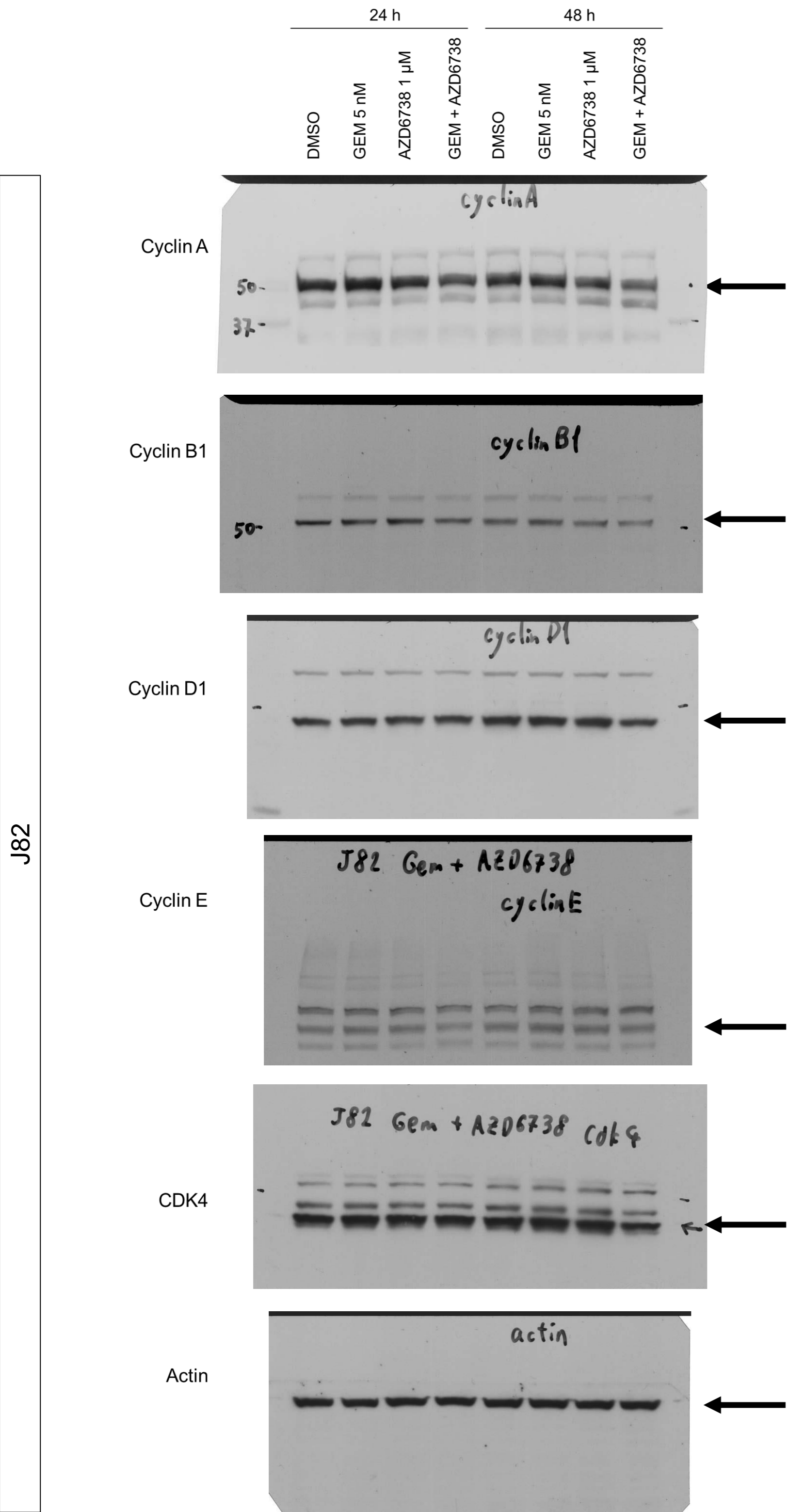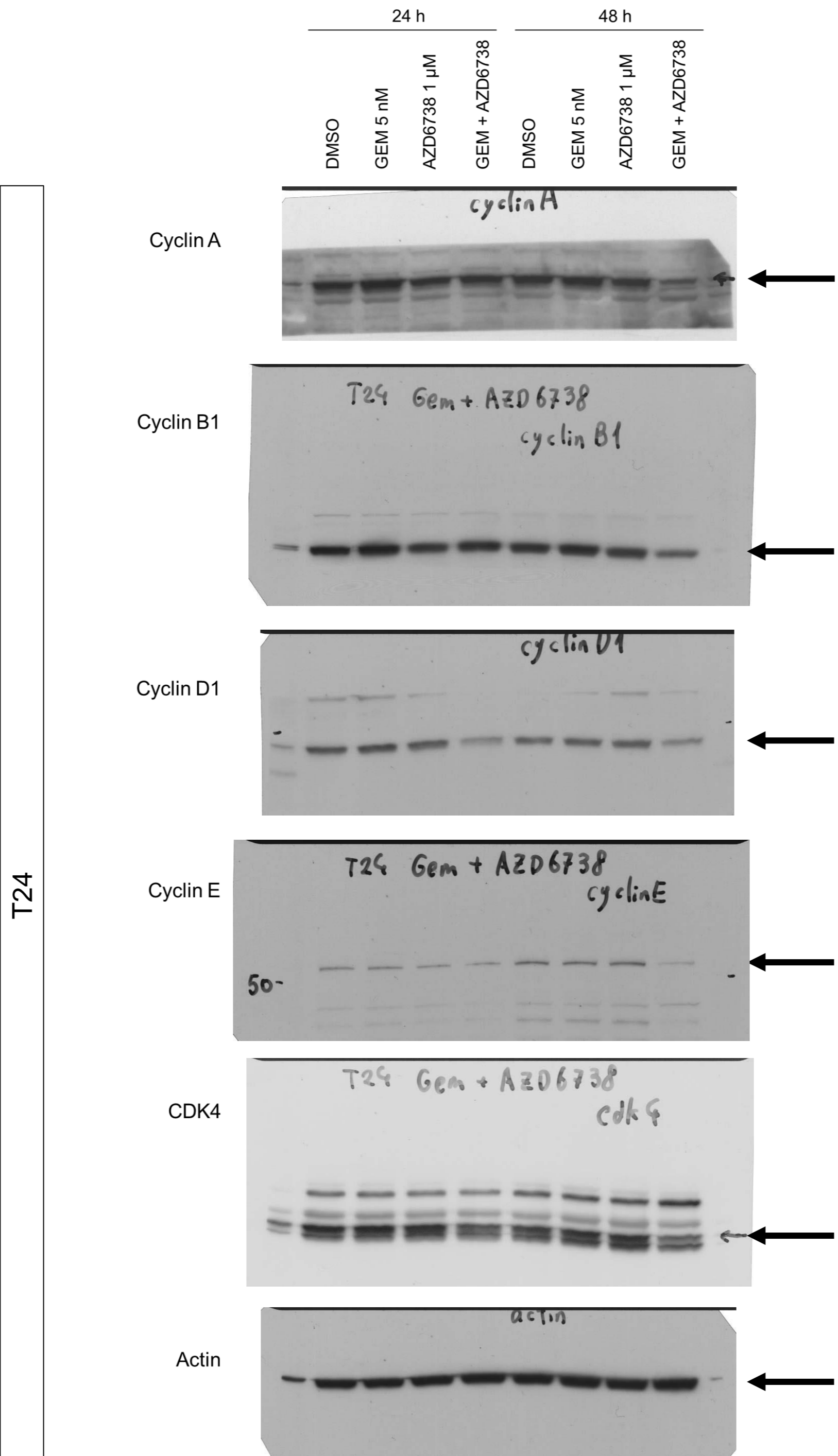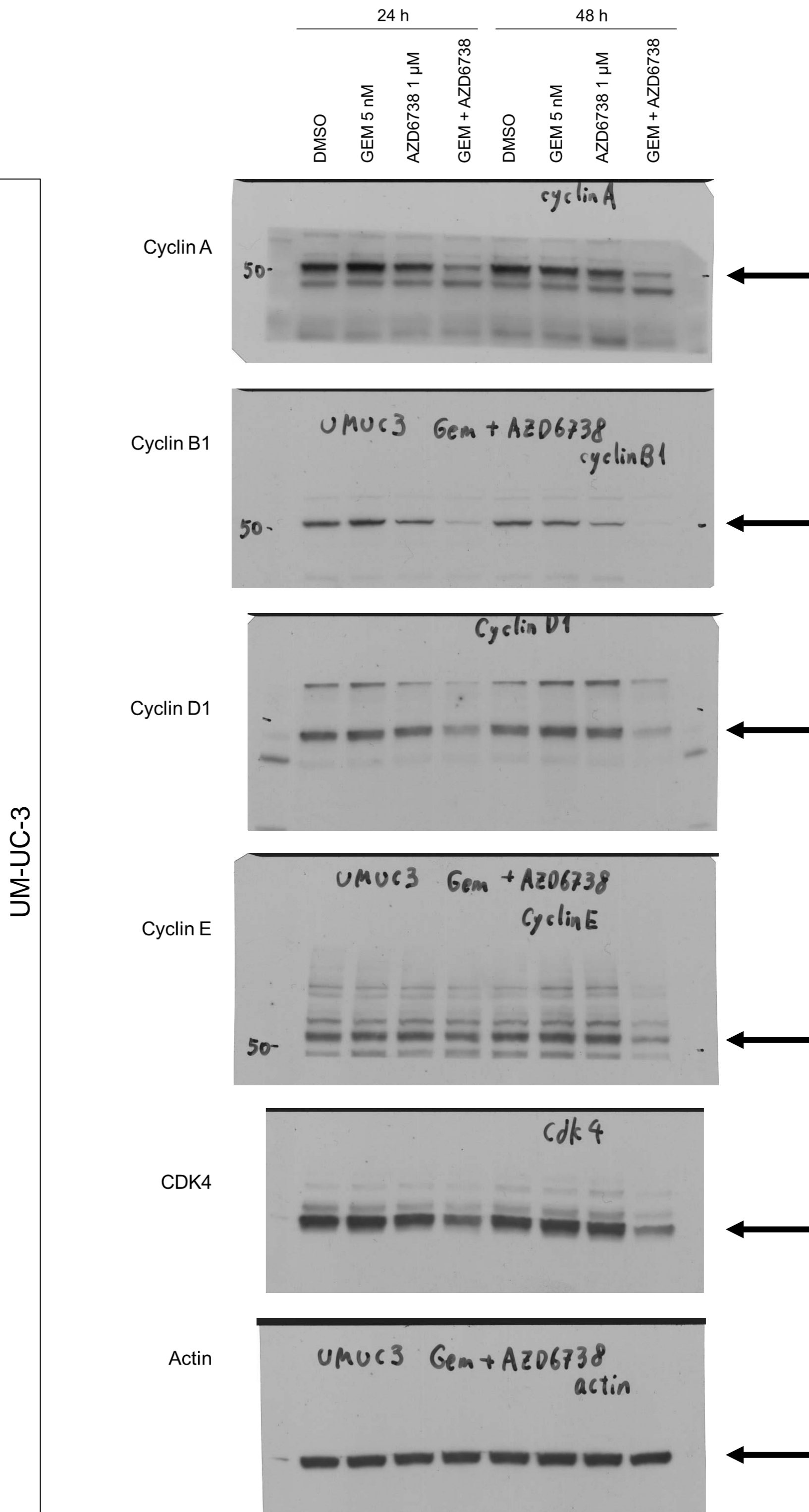

Figure 3B

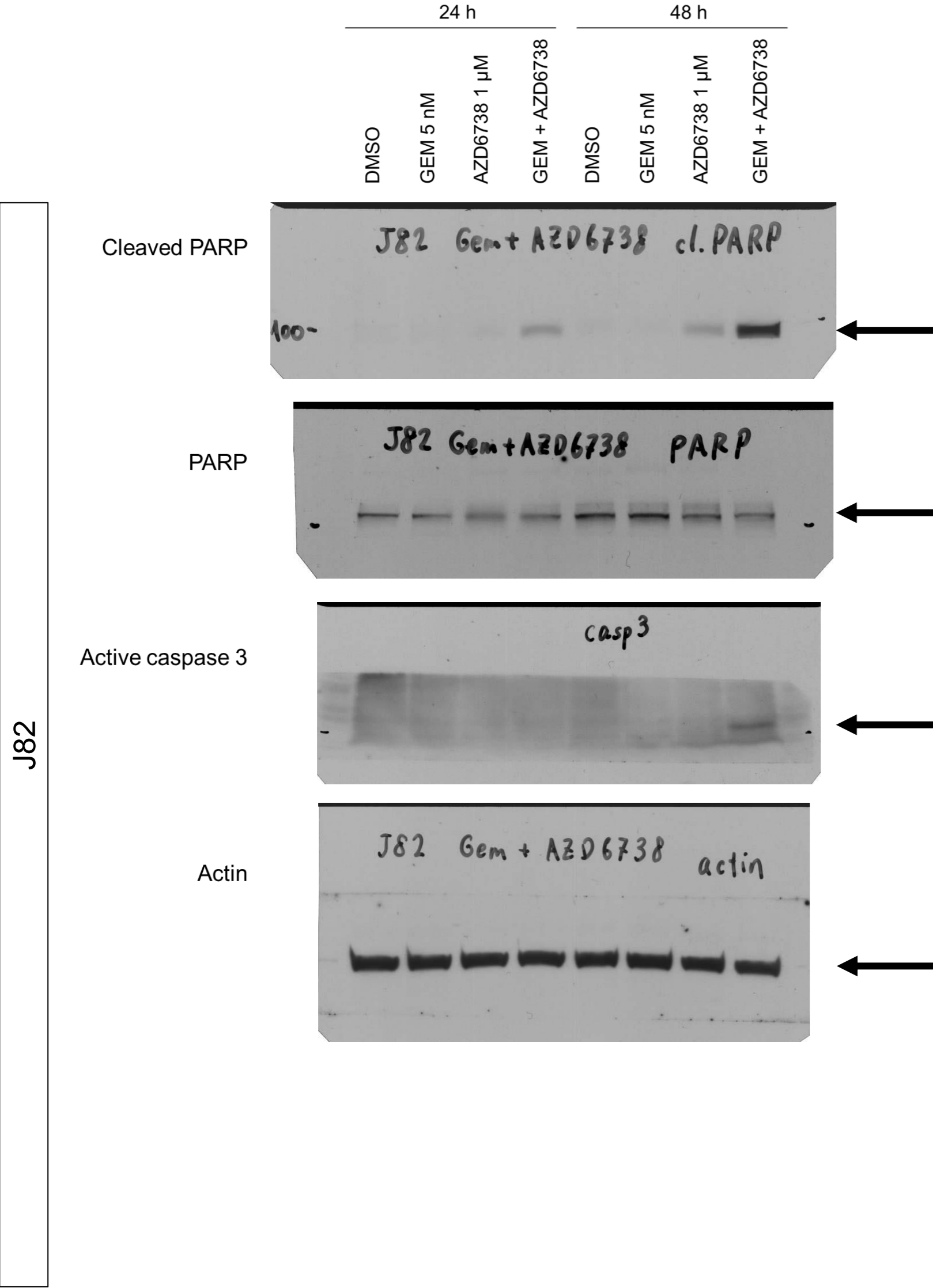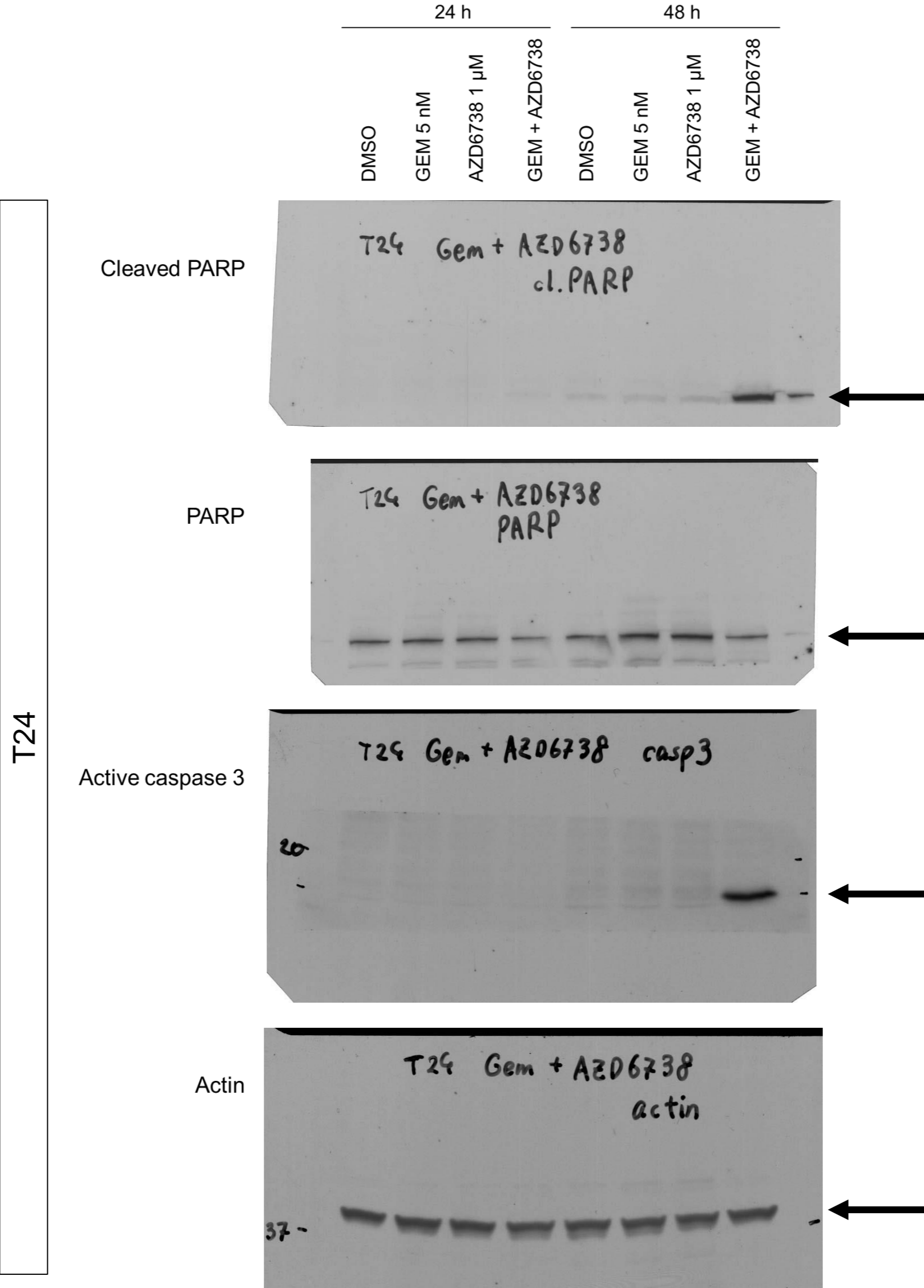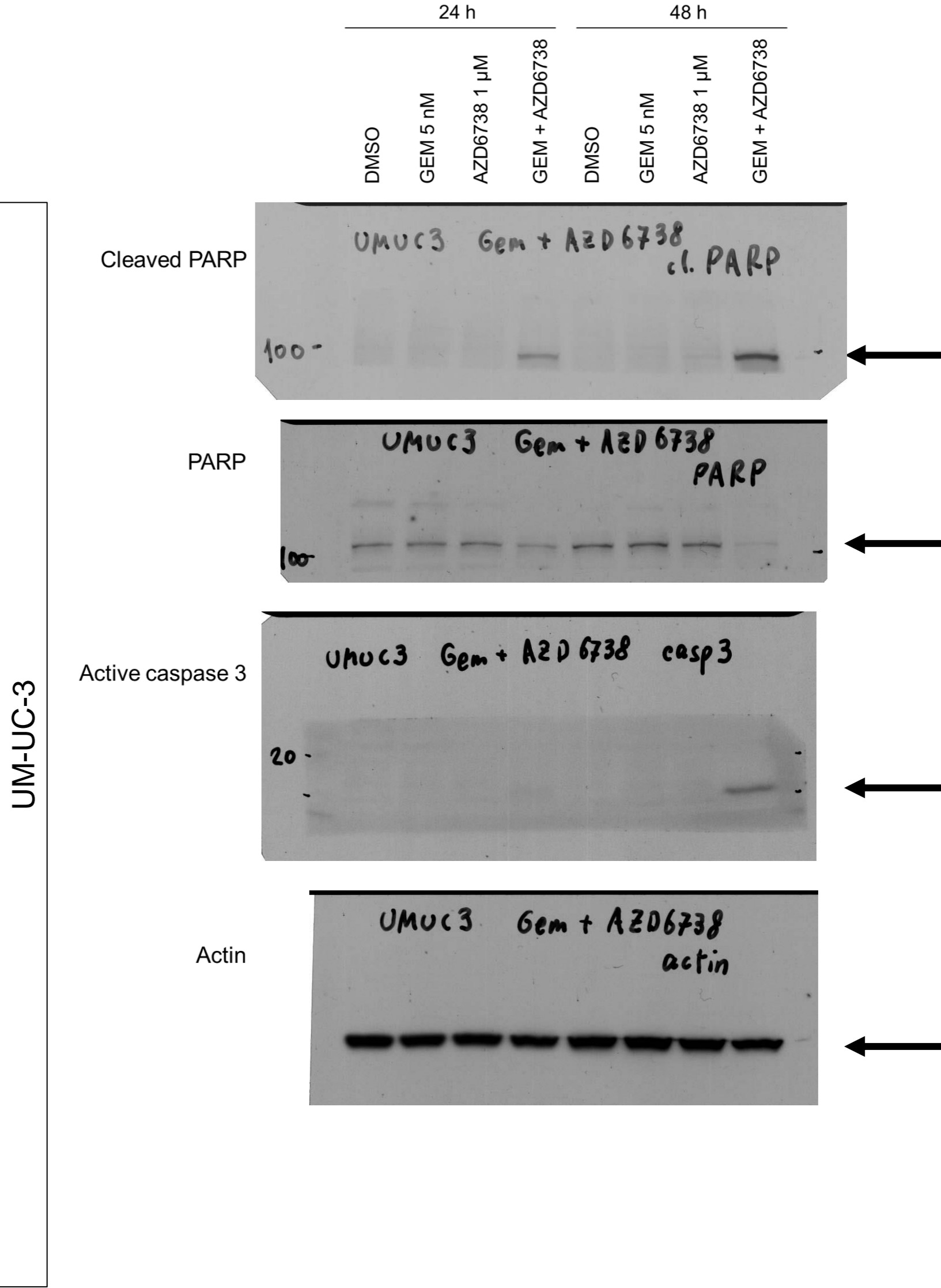

Figure 4A

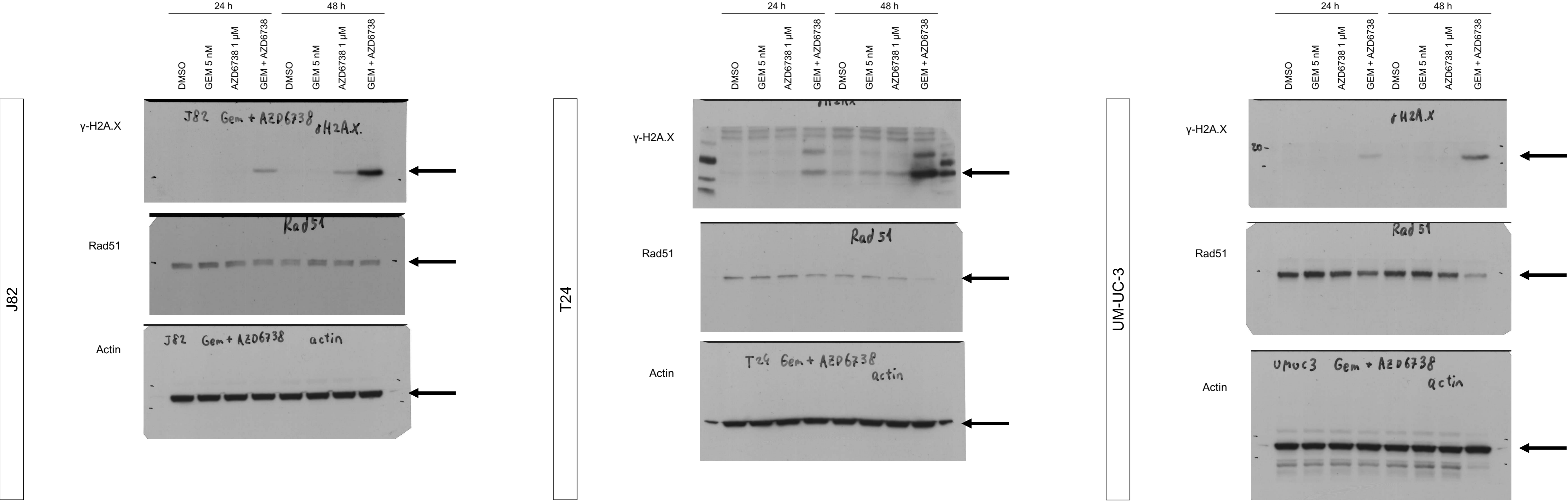

Figure 4B

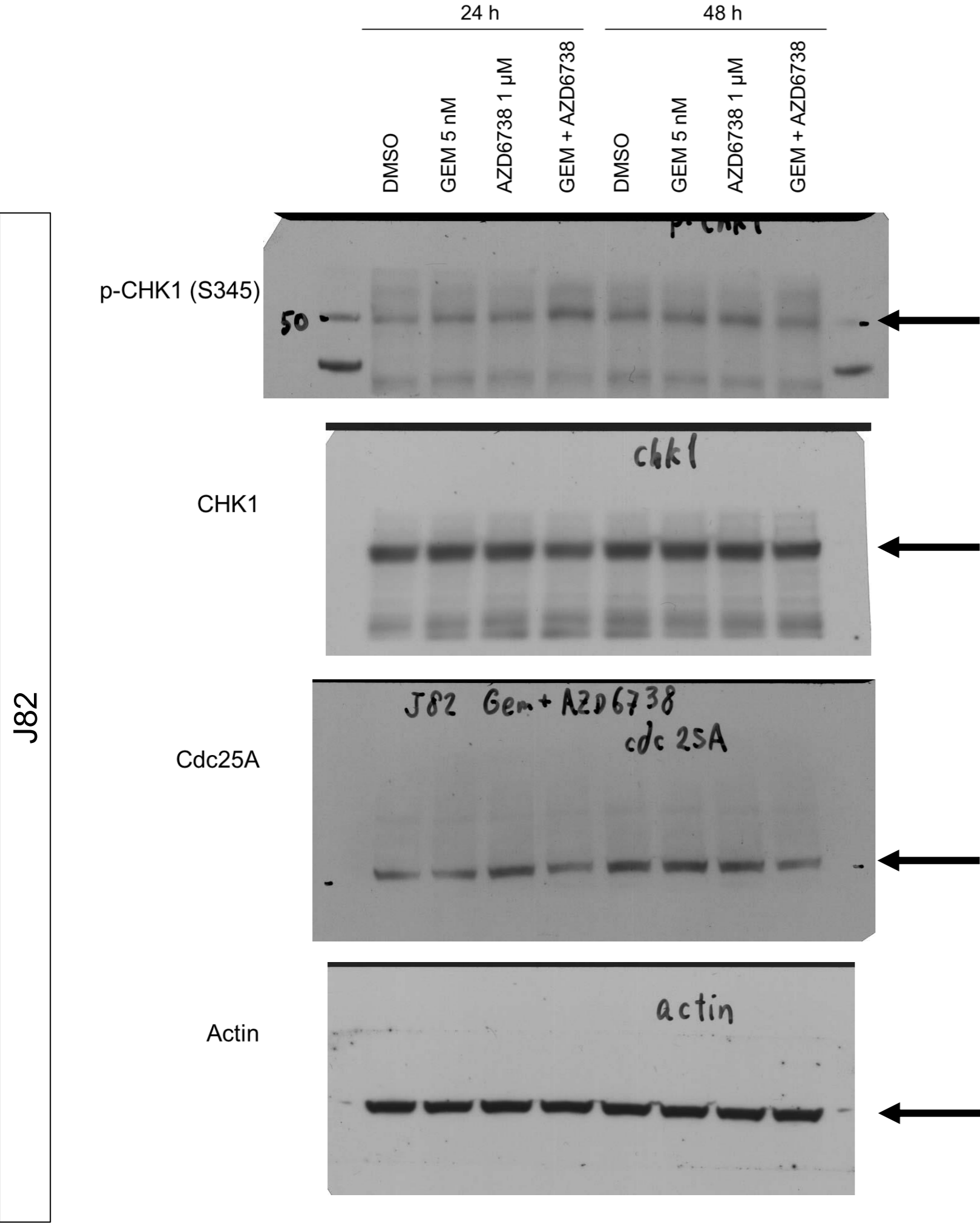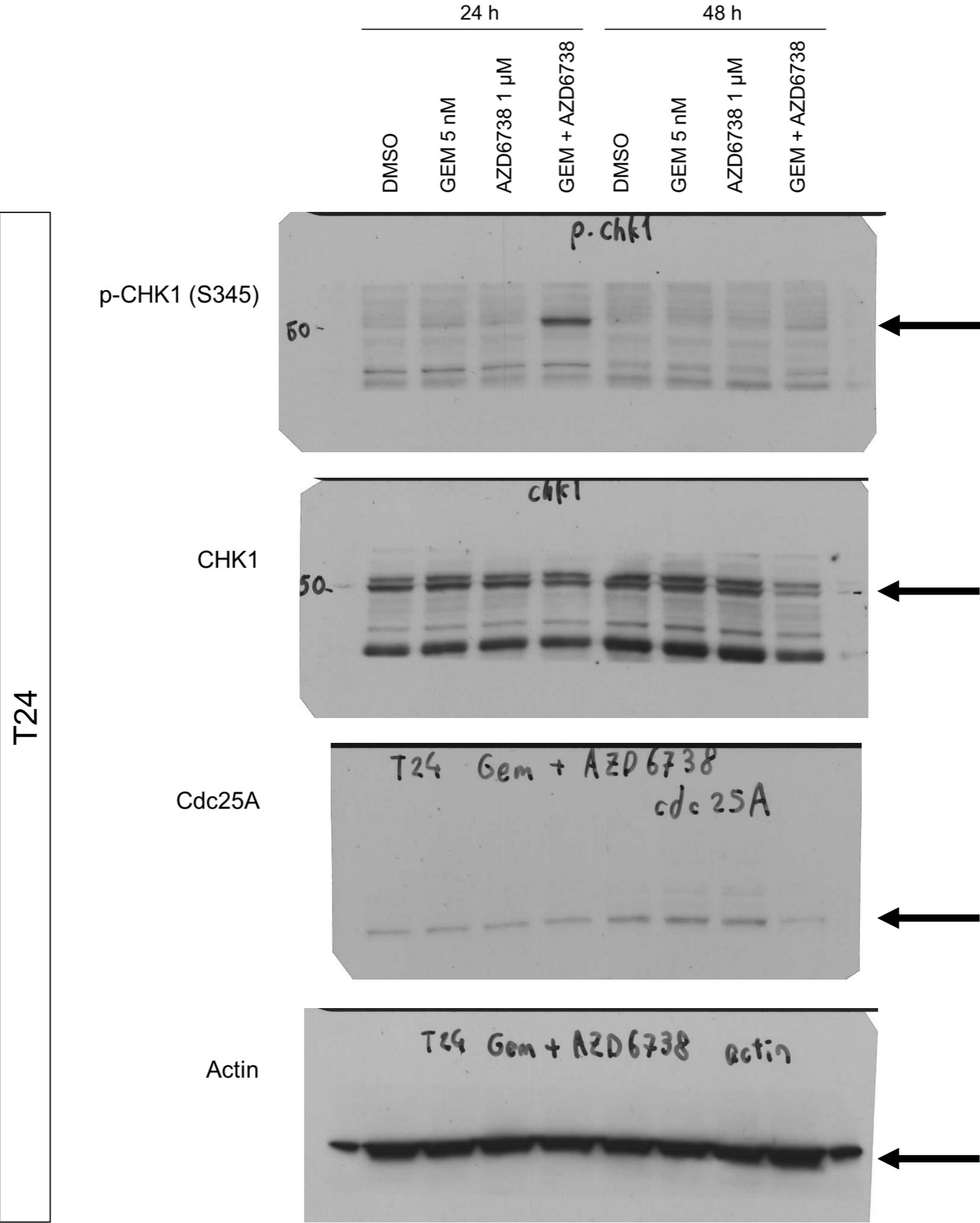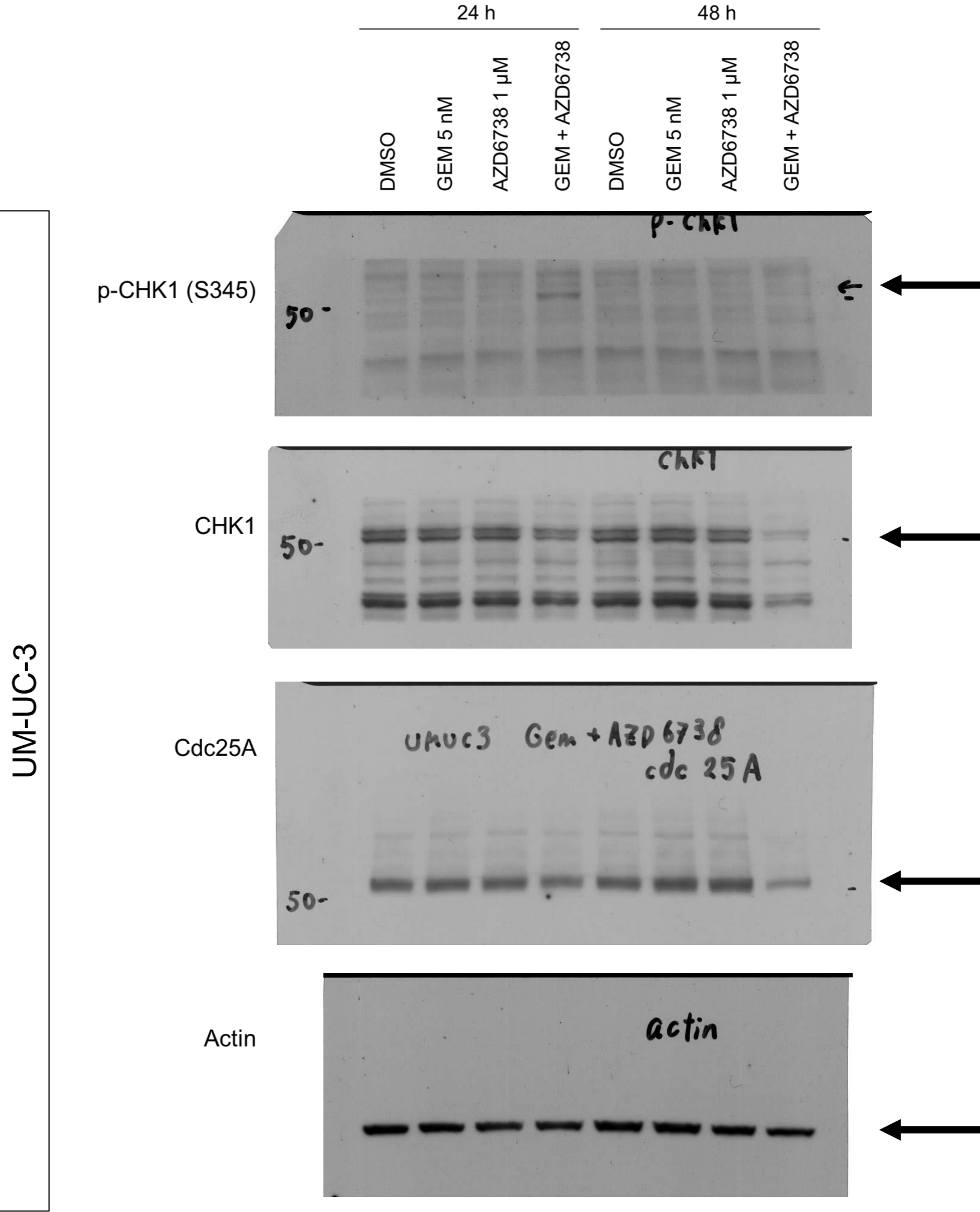

Supplement: S5 Fig — Arrows indicate cropped bands. Note that the membranes were cut before probing. (PDF) [file pone.0266476.s005.pdf]
